# Mobile Mental Health Units in rural Greece: successful and cost-effective model

**DOI:** 10.1192/bji.2025.10052

**Published:** 2026-08

**Authors:** Vaios Peritogiannis, Maria Samakouri, Alexandros Vgontzas

**Affiliations:** 1 Head of Department, Mobile Mental Health Unit of the Prefectures of Ioannina and Thesprotia, Society for the Promotion of Mental Health in Epirus, Ioannina, Greece; 2 Professor of Psychiatry, Head of Department, Medical School, Democritus University of Thrace, Alexandroupolis, Greece; 3 Professor of Psychiaty, Head of Department, Mobile Mental Health Unit, Department of Psychiatry and Behavioral Sciences, School of Medicine, University of Crete, Heraklion, Greece

**Keywords:** Community mental health, Greece, mobile mental health units, rural areas, severe mental illness

## Abstract

The Greek model of mental healthcare delivery in rural and remote low-resourced areas is based on Mobile Mental Health Units (MMHUs). MMHUs are low-cost, interdisciplinary, generic community psychiatric services that collaborate closely with the primary healthcare system and local social services. MMHUs may be effective in the treatment of patients with severe and persistent mental illness and cost-effective in the treatment of patients with common mental disorders. The Council of Europe categorised this model as an effective community-based initiative. Major challenges derive from the difficulties MMHUs run by public general hospitals face in recruiting and retaining personnel.

People living in rural and remote areas may lack access to effective and affordable mental healthcare, due to disparities between rural and urban populations. The practice of mobile mental healthcare has emerged in response to the needs of underserved communities.^1^ In several regions in rural Greece, mental healthcare is delivered by locally based generic community mental health teams, known as Mobile Mental Health Units (MMHUs).^2^ The aim of the present paper is to present the model of MMHUs in rural Greece; to examine its relevance and its success for mental healthcare and policy in remote and underserved areas; and to highlight the challenges in delivering mental health services in such areas.

## The Greek rural context

Greece, at least its mainland, is a largely mountainous country. Consequently, access to healthcare may be difficult, particularly in winter, for a substantial proportion of the population residing in rural areas. Population density is low in these areas; rates of poverty and unemployment are high, whereas the population is steadily ageing.^3^ Furthermore, many of the numerous islands in the Greek territory lack mental health services and have limited access to centrally located services. Accordingly, it has been suggested that rural areas in Greece may not receive adequate mental healthcare.^4^

## Basic characteristics of MMHUs

MMHUs in rural Greece are low-cost, generic community psychiatric services that deliver evidence-based mental healthcare and promote mental health in remote and deprived areas that lack access to such care.^2^ They are publicly funded services, mostly run by non-profit, non-governmental organisations (NGOs) or by general or university hospitals. MMHUs consist of an interdisciplinary team of mental health specialists (such as psychiatrists, psychologists, nursing staff, social workers and health visitors) that collaborates closely with the primary healthcare system, social services such as ‘Assistance at Home’, local community organisations and authorities such as the church, police, and cultural organisations, and local town/village authorities.^5^ These units deliver community-oriented treatment and prevention services for the whole range of mental disorders to adults and on several occasions to children and adolescents in their catchment area. Concerning adults, emphasis is placed on patients with severe and persistent mental illness, such as schizophrenia-spectrum disorders, bipolar disorder or prevalent mood disorders – that is, depression in the geriatric population.^2^ MMHUs use the infrastructures of the well-established primary healthcare network in rural areas and may visit certain patients at home, when needed.

## The current state of MMHUs in rural Greece

The most recent (2019) recorded number of MMHUs is 20,^6^ but over the years the operation of some MMHUs, all run by public hospitals, has ceased, reflecting the widespread difficulties faced by public health services in Greece. Fig. 1 depicts the areas that are covered by MMHUs across Greece.

**Fig. 1:**
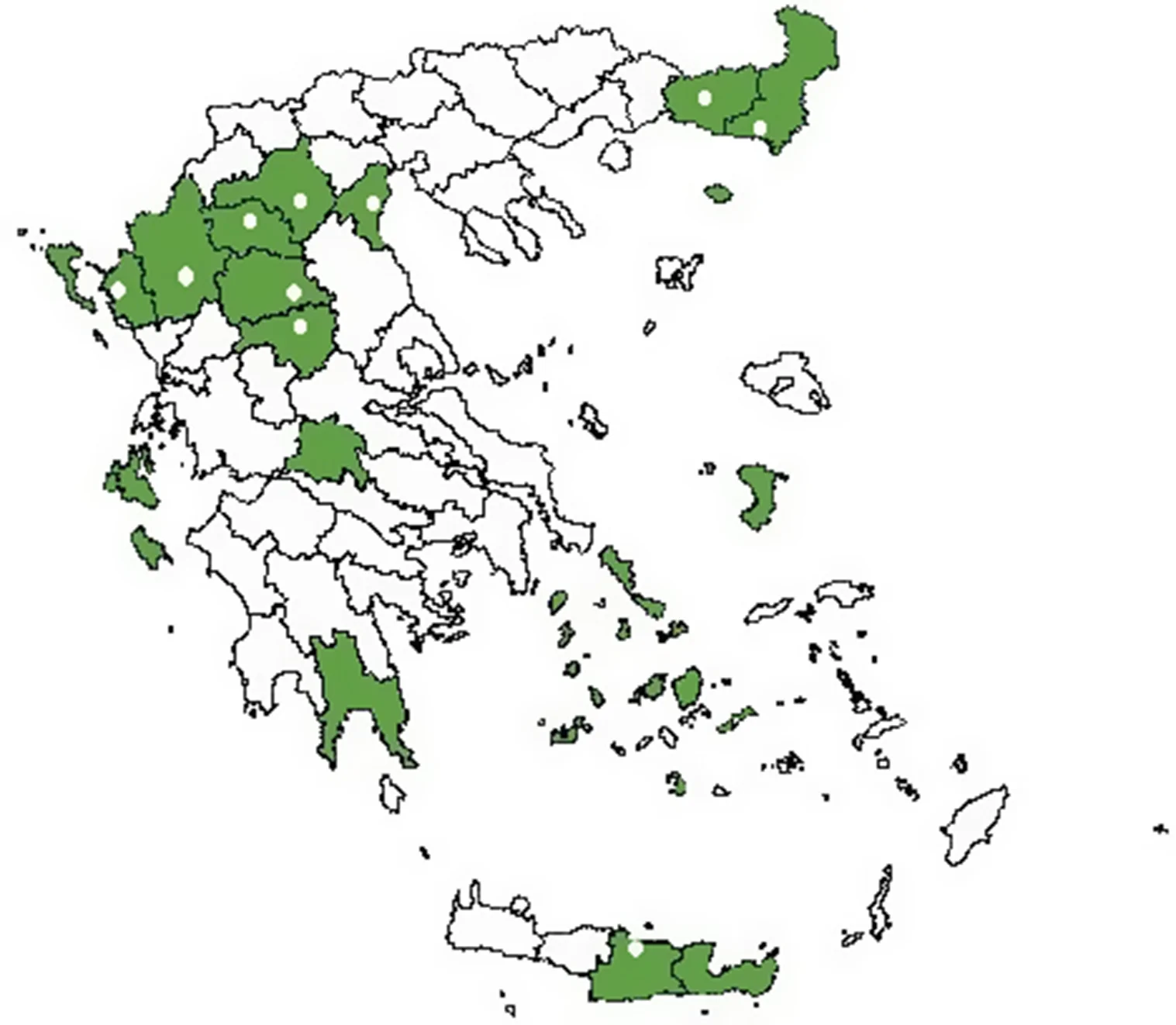
Map of Greece. Areas that are covered by Mobile Mental Health Units (MMHUs) are highlighted. These areas comprise a population of >1 080 000 residents. Only MMHUs that deliver services for the whole adult population are included (*n* = 17).

There may be significant differences among MMHUs in terms of clientele, staffing and provided services.^7^ Several MMHUs do not deliver services for children and adolescents, while only a few deliver the recently launched modified assertive community treatment (ACT) service. Moreover, MMHUs on islands may be the only available local mental health services, also serving semi-urban areas. Such differences partly derive from geographical areas. Notably, some islands in the Aegean Sea, Eastern Greece have accepted a large number of refugees and immigrants in recent years; accordingly, MMHUs in those islands have undertaken the mental health care of this vulnerable population.^8^ Another possible explanation for the observed differences is that the foundation of these units is not based on an objective assessment of the mental health needs of the population residing in a specific catchment area.

## Effectiveness and cost-effectiveness

A recent report summarised the literature on the effectiveness of MMHUs in terms of reduction of hospitalisations and length of hospital stay of patients with severe and persistent mental illness, and reduction of disability and cost-effectiveness in patients with common mental disorders. Given that current evidence is based on only a few MMHUs, further research with multicentre studies and larger samples of patients is warranted to establish the effectiveness and cost-effectiveness of the MMHUs.^9^

## Challenges and limitations

Working in rural areas may not be adequately appreciated and may be less rewarding for mental health professionals, who may prefer to work in urban areas. Indeed, Greece has major imbalances in distribution and availability of human resources for health. For instance, there is a shortage of children and adolescent psychiatrists in the public sector, as well as unequal distribution, particularly in rural areas.^10^ Delivery of services for children and adolescents may not always be feasible in the context of an MMHU.

The elderly constitute a large proportion of adults in rural Greece, and many suffer from major disorders such as depression and dementia complicated by low income, social isolation and loneliness, primarily in women, and lack of community resources. In addition to continuous training of the MMHUs’ workforce in psychogeriatrics, a closer and more effective collaboration with the local network of Primary Care Health Centers is warranted to improve the current level of care in this population.

The stigma attached to mental illness is another challenge faced by MMHUs. The fear of being perceived as mentally ill is stronger in rural, remote, low-density population areas given that everyone knows everyone and confidentiality and/or privacy is not a given. This fear leads the families of severely mentally ill people to seek treatment in the private sector in a nearby city to secure anonymity. A major disadvantage of this approach is that after the first couple of visits to a private physician, regular follow-up is unaffordable for most patients and their families. Hopefully, the long uninterrupted presence of MMHUs and the application of several methods to educate/sensitise the public about mental illness and the success of current treatments will reduce the burden of stigma.

Finally, MMHUs run by public general hospitals may have difficulties in recruiting, retaining and replacing personnel, which may result in discontinuation of the operation of an MMHU.

## Implications and relevance for clinical practice and mental health care policy

The pursuit of high-quality, cost-effective services is relevant in remote and underserved areas. The paradigm of the MMHUs may be a good fit in such areas, even in developed countries, given the mental health disparities between the urban, highly resourced settings and the rural, low-resourced ones. MMHUs could optimise mental healthcare delivery in communities which have limited access to mental healthcare by overcoming barriers deriving from travel constraints, patient–provider communication, financial issues and complex administrative processes. Accordingly, the Council of Europe, in a report on good practices for the promotion of voluntary measures in mental health services, categorised the Greek model of MMHUs as an effective community-based initiative.^11^

Despite major investments in highly specialised and adequately resourced services, such as the early intervention services for severe mental illness or ACT in Western countries, generic community-based services should not be overlooked by policymakers. In rural areas with low-resourced settings, MMHUs may be the best option for delivering comprehensive, evidence-based care. Along with the establishment of complex and highly resourced interventions, the Greek state should adequately fund MMHUs in order to expand care in most rural, remote and insular areas.

## Some considerations

Several points need to be considered in the discussion of community mental health services in rural areas. First, although several core characteristics such as isolation, small towns, low population density and poverty are largely associated with rural residency, a standardised definition of ‘rural’ is still lacking.^12^ This may limit generalisability of research findings across studies and countries. Additionally, although community mental health services were not designed to be physician-centered, the role of psychiatrists in the operation of MMHUs is pivotal. Finally, the usefulness of modern technology such as telepsychiatry, supplementary to in-person delivery of services, should be examined.

## Conclusions

MMHUs are a low-cost model of mental healthcare delivery that advances health equity by enabling access to care in populations living in resource-limited rural and remote areas. Although this model is yet to be fully evaluated, it appears to be effective in the treatment of the most severe and/or prevalent mental disorders. MMHUs still face many challenges, including lack of adequate child psychiatric services, that may undermine their operation and must be addressed by the Greek state. This model of care seems worthy of expansion in most rural areas and may also be suitable for urban settings. The modified hybrid ACT model that is being run by some MMHUs should also be evaluated and possibly expanded as well. More research is needed, with the collaboration of as many MMHUs as possible, for the conduct of multicentre studies that could determine generalisability and applicability of results.

## Data Availability

Data availability is not applicable to this article as no new data were created or analysed in this study.
